# An inflammatory myofibroblastic tumor exhibiting immunoreactivity to KIT: a case report focusing on a diagnostic pitfall

**DOI:** 10.1186/1477-7819-12-186

**Published:** 2014-06-18

**Authors:** Tatsuki R Kataoka, Nobuhiro Yamashita, Ayako Furuhata, Masahiro Hirata, Takaki Ishida, Ichiro Nakamura, Seiichi Hirota, Hironori Haga, Eiji Katsuyama

**Affiliations:** 1 Department of Diagnostic Pathology, Kyoto University Hospital, Sakyo-ku, Kyoto 606-8507, Japan; 2 Department of Pathology and Laboratory Medicine, Kobe City Medical Center West Hospital, Kobe, Hyogo 653-0013, Japan; 3 Department of Urology, Kobe City Medical Center West Hospital, Kobe, Hyogo 653-0013, Japan; 4 Department of Surgical Pathology, Hyogo College of Medicine, Nishinomiya, Hyogo 663-8501, Japan

**Keywords:** Anaplastic lymphoma kinase, Differential diagnosis, FISH, Gastrointestinal stromal tumor, Inflammatory myofibroblastic tumor, KIT, Urinary bladder

## Abstract

Inflammatory myofibroblastic tumors (IMTs) and gastrointestinal stromal tumors (GISTs) are both spindle cell tumors, and occur rarely in the wall of the urinary bladder. In general, immunostaining allows differentiation of IMTs and GISTs. Most IMTs are positive for anaplastic lymphoma kinase (ALK) and negative for KIT, whereas most GISTs are ALK-negative and KIT-positive. Here, we describe a case of a spindle cell tumor in the wall of the urinary bladder. The spindle cells were positive for both ALK and KIT, and it was thus difficult to determine whether the tumor was an IMT or a GIST. We eventually diagnosed an IMT, because *ALK* gene rearrangement was confirmed by fluorescent in-situ hybridization. Cytoplasmic staining for KIT and the absence of other GIST markers, including DOG1 and platelet-derived growth factor α, indicated that the tumor was not a GIST. Therefore, IMTs should be included in the differential diagnosis of spindle cell tumors, even those that are KIT-positive.

## Background

Inflammatory myofibroblastic tumors (IMTs) are spindle cell tumors sometimes considered to exhibit borderline malignancy [1]. These tumors are characterized by the infiltration of inflammatory cells, including lymphocytes, plasma cells, and eosinophils [1] and can occur at any site in the body, including the urinary bladder [2,3]. Approximately half of all IMTs exhibit anaplastic lymphoma kinase (*ALK*) gene rearrangements [4], which are considered to be an IMT marker, as are expression of vimentin, muscle-specific actin, and calponin [5-7]. The majority of IMTs do not express S-100, KIT, or CD34 [5-7].

A gastrointestinal stromal tumor (GIST) is another form of spindle cell tumor [8] that rarely occurs in the urinary bladder [9,10]. Most GISTs exhibit gain-of-function mutations in the *KIT* gene and express KIT protein [11,12]. KIT is a receptor-type tyrosine kinase [13] and is thus usually present in membranes, but it may also be expressed in the cytoplasm. Some GISTs lacking *KIT* mutations have mutations in the platelet-derived growth factor receptor α (*PDGFRα*) gene [14]. In such cases, PDGFRα serves as a GIST marker [14]. The *DOG1* gene is also considered to be a marker of GIST [15].

Here, we describe a spindle cell tumor that developed in the wall of the urinary bladder. Immunostaining showed that the tumor cells were positive for both ALK and KIT expression, and it was thus difficult to distinguish whether the tumor was an IMT or GIST.

## Case presentation

### Methods

#### ***Immunostaining***

The antibodies used are listed in Tables 1 and 2. Cytological specimens were fixed in 100% methanol, and pathological specimens were fixed in formalin and paraffin-embedded. Both types of specimen were stained, as described in a previous report [16], in addition to ALK immunohistochemical staining of pathological specimens, which was performed using the intercalated antibody-enhanced polymer (iAEP) method [17].

**Table 1:** Antibodies used for immunostaining in immunocytochemical analyses.

**Antigen**	**Clone**	**Dilution**	**Source**	**Pretreatment**
SMA	1A4	Prediluted	Nichirei	None
Desmin	D33	Prediluted	Nichirei	None
Pan-cytokeratin	AE1/AE3	Prediluted	Nichirei	None
KIT	Polyclonal	1:25	DAKO	None
ALK	5A4	Prediluted	Nichirei	None

ALK, anaplastic lymphoma kinase.

**Table 2:** Antibodies used in immunostaining in immunohistological analyses.

**Antigen**	**Clone**	**Dilution**	**Source**	**Pretreatment**
**SMA**	1A4	Prediluted	Nichirei	None
**Desmin**	D33	Prediluted	Nichirei	Microwave (10 min)
**Pan-cytokeratin**	AE1/AE3	Prediluted	Nichirei	Protease
**KIT**	Polyclonal	1:25	DAKO	Microwave (10 min)
**ALK**	5A4	1:50	Nichirei	Boiling (30 min)*
**PDGFR**	Polyclonal	1:50	Santa Cruz	Boiling (60 min)
**DOG1**	SP31	1:50	Nichirei	Boiling (60 min)
**IgG**	EPR4421	1:50	Epitomics	Boiling (30 min)
**IgG4**	HP6025	1:50	Nichirei	Boiling (60 min)

* The iAEP method was required to obtain the signals. ALK, anaplastic lymphoma kinase; IgG, immunoglobulin G; PDGFR, platelet-derived growth factor receptor.

#### ***KIT mutation analysis***

The DNA was extracted from pathological specimens. Exons 11 and 17 of the *KIT* gene were amplified by PCR and used for direct sequencing [18].

#### ***Fluorescent in-situ hybridization (FISH) examination of ALK***

To explore *ALK* gene rearrangements, fluorescent *in-situ* hybridization (FISH) was performed using a Vysis ALK Break Apart FISH Probe kit (Abbott Molecular, Des Plaines, IL, USA) [19]. Briefly, unstained sections (4 μm thick) were subjected to hybridization according to the manufacturer’s protocol. *ALK* rearrangement was considered present when over 15% of the tumor cells displayed orange and green hybrid signals or an orange signal alone.

#### ***Clinical history***

**Figure 1:**
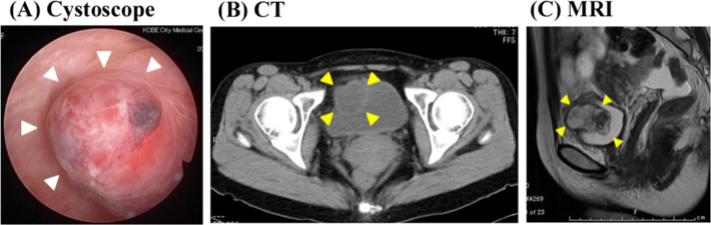
**Cystoscopy, computed tomography, and magnetic resonance imaging findings. (A)** Cystoscopy, **(B)** computed tomography, and **(C)** magnetic resonance imaging. White and yellow arrowheads indicate the submucosal tumor in the urinary bladder.

Our patient was a 31-year-old woman initially admitted to another clinic because of painful urination and subsequently transferred to our hospital for further examination. First, we performed a cytological study of her urine and a cystoscopic examination. The cytological study did not yield any specific findings, but cystoscopy detected a submucosal tumor lying beneath the normal mucosa (Figure 1A). Computed tomography and magnetic resonance imaging identified a mass 4.5 cm in diameter in the right lateral wall of the urinary bladder (Figure 1B,C). Transurethral resection of the bladder tumor (TUR-Bt) was performed to obtain a diagnosis. Spindle cells were collected during TUR-Bt, but they did not yield any diagnostic information because of heat denaturation.

**Figure 2:**
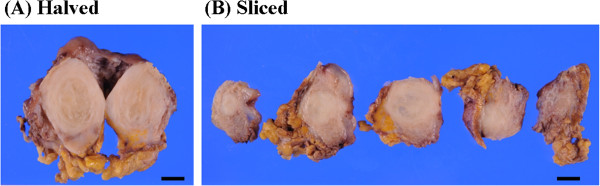
**Macroscopic findings. (A)** Halved specimens. **(B)** Sliced specimens. Bar, 1 cm.

**Figure 3:**
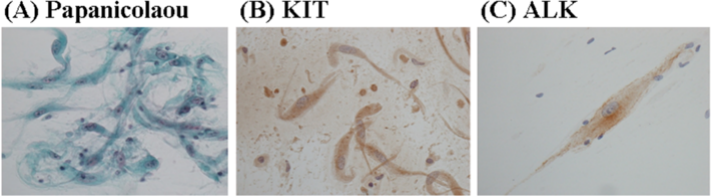
**Cytological findings. (A)** Papanicolaou staining. **(B)** KIT staining using a rabbit polyclonal antibody purchased from DAKO. **(C)** ALK staining using a monoclonal antibody purchased from Nichirei. All photographs were obtained at 400× magnification.

**Figure 4:**
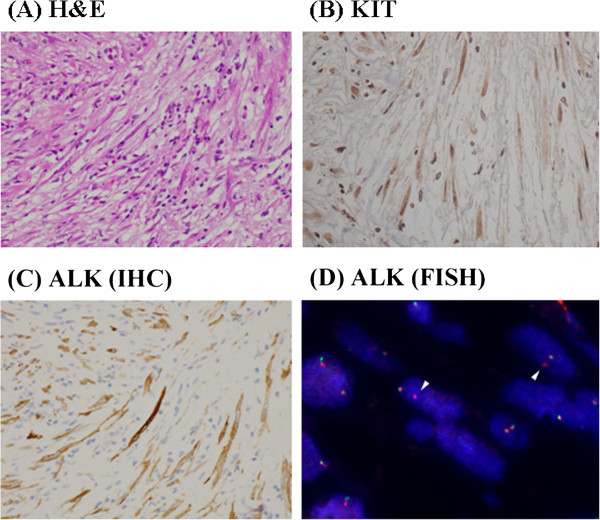
**Histological findings. (A)** H & E staining. **(B)** KIT staining using a rabbit polyclonal antibody purchased from DAKO. **(C)** ALK staining using a monoclonal antibody purchased from Nichirei. **(D)** FISH analysis using the Vysis ALK Break Apart FISH Probe kit. White arrowheads indicate signals positive for *ALK* gene rearrangements. All photographs were obtained at 200× magnification.

We surgically resected the mass (Figure 2), which was located in the muscular layer of the urinary bladder, and was thus not contiguous with the mucosa. Imprint cytology was performed during resection (Figure 3A) and revealed numerous spindle cells with an abundance of cytoplasm and low nuclear atypia, admixed with infiltrating lymphocytes. Immunocytochemically, the spindle cells were negative for smooth muscle actin and desmin, but an anti-KIT antibody yielded a positive reaction (Figure 3B). However, the cells were also positive for ALK (Figure 3C). Histologically, a spindle cell tumor that was immunohistochemically positive for both KIT and ALK was also identified (Figure 4A-C). The fact that the nuclear atypia was of low grade and that a malignant component was lacking allowed us to rule out sarcomatoid cancer. We also excluded leiomyosarcoma and rhabdomyosarcoma because the tumor was negative for smooth muscle actin and desmin. In addition, staining for IgG and IgG4 indicated that the tumor was not an IgG4-related inflammatory pseudotumor, because IgG4-positive cells were very rare. A GIST was considered in differential diagnosis. We found no mutation in the *KIT* gene. The tumor cell cytoplasm stained weakly for KIT, which is unusual (but not unknown) for GIST tumors [11,12]. In addition, the cells were negative for other GIST markers (that is, PDGFRα and DOG1) [14,15]; therefore, we ruled out GIST. To evaluate the *ALK* gene status, we performed FISH analysis using the Vysis *ALK* Break Apart FISH Probe kit and found that approximately 20% of the signals indicated *ALK* gene rearrangement (Figure 4D). Thus, we diagnosed the tumor as an IMT.

Our patient is alive and well, without recurrence, 3 years after resection.

## Discussion

Most submucosal tumors in the wall of the urinary bladder are spindle cell tumors [20,21]. The spindle cell tumor in our patient expressed ALK. To the best of our knowledge, only one previous report examined the ALK status of GISTs and found that such tumors were ALK-negative [22]. However, more evidence is required to rule out a GIST, even if a spindle cell tumor is positive for ALK. *ALK* rearrangement is associated with the pathogenesis of various malignancies, including anaplastic large-cell lymphoma, non-small cell lung cancer, and neuroblastoma [23]. In addition, *ALK* rearrangement, associated with an altered enzyme expression level, is detected in approximately half of IMTs [4-7]. In the current case, we found that a significant proportion of *ALK* genes were split (Figure 4D).

Morphological discrimination between IMT and GIST was nearly impossible in the present case, but we found that cytological examination was useful in this context. It was feasible to stain cytological specimens for ALK, and no pretreatment was required. However, we had to use a sophisticated method (iAEP method [19]) to stain the histological specimens (Table 1). Thus, cytological analysis using ALK immunostaining might be useful in differentiating an IMT from a GIST.

A variety of spindle cell tumors exhibit KIT positivity, including mesothelioma [24], leiomyosarcoma [24], clear cell sarcoma [24], rhabdomyosarcoma [24], synovial sarcoma [24], angiomyolipoma [25,26], bladder urothelial carcinoma [24,27], and malignant melanoma [27]. Therefore, KIT immunopositivity alone cannot differentially diagnose a GIST among spindle cell tumors. In this case, the KIT staining pattern and the lack of PDGFRα and DOG1 immunoreactivity suggested that a GIST was unlikely.

Eventually, we diagnosed the tumor as a KIT-positive IMT. Such tumors are rare, with only two cases reported in the English-language literature [2]. Interestingly, both tumors were in the urinary bladder [2]. Therefore, KIT positivity alone might not be adequate to diagnose a GIST, especially if the tumor is in the urinary bladder.

## Conclusions

A possibility of IMT should be included in the differential diagnosis of KIT-positive spindle cell tumors, especially those in the urinary bladder. Molecular exploration of *ALK* rearrangements and *KIT* mutations may be required to diagnose KIT-positive spindle cell tumors.

## Consent

Our patient kindly provided written informed consent for publication of this case report and the accompanying images.

## Abbreviations

ALK: anaplastic lymphoma kinase; FISH: fluorescent in-situ hybridization; GIST: gastrointestinal stromal tumor; H & E: hematoxylin and eosin; iAEP: intercalated antibody-enhanced polymer; IgG: immunoglobulin G; IMT: inflammatory myofibroblastic tumor; PCR: polymerase chain reaction; PDGFRα: platelet-derived growth factor receptor α; TUR-Bt: transurethral resection of bladder tumor.

## Competing interests

The authors declare that they have no competing interests.

## Authors’ contributions

TRK, SH, HH, and EK conceived and designed the study. NY and MH performed the immunocytochemical and immunohistochemical assays. AF performed the FISH analysis. SH screened for *KIT* mutations. TI and IN collected the clinical data. TRK, NY, and EK assembled the data. TRK and HH wrote the manuscript. All authors approved the final manuscript.
